# Crack Cocaine and Infectious Tuberculosis

**DOI:** 10.3201/eid1409.070654

**Published:** 2008-09

**Authors:** Alistair Story, Graham Bothamley, Andrew Hayward

**Affiliations:** Health Protection Agency, London, UK (A. Story); Homerton Hospitals National Health Service Foundation Trust, London (G. Bothamley); University College London Centre for Infectious Disease Epidemiology, London (A. Hayward)

**Keywords:** Tuberculosis, crack cocaine, street drugs, dispatch

## Abstract

We hypothesize that crack cocaine is independently associated with smear-positive tuberculosis (TB). In a case–control study of TB in London, 19 (86%) of 22 crack cocaine users with pulmonary TB were smear positive compared with 302 (36%) of 833 non–drug users. Respiratory damage caused by crack cocaine may predispose drug users to infectivity.

Tuberculosis (TB) has reemerged as a public health problem in London, and drug users are at high risk of contracting and spreading the disease (*1*). The United Kingdom has seen a substantial increase in the prevalence of drug use in the past decade, particularly crack cocaine use (*2*). Numbers of crack cocaine users assessed while in police custody in London increased 3-fold from 1993 through 2003 (*3*). There are an estimated 46,000 crack cocaine users in London; most also use opiates (*4*). Evidence to directly link risk for TB with crack cocaine use is lacking, although an association with tuberculin positivity has been shown. Increased exposure risk is considered largely attributable to social and lifestyle factors including homelessness, imprisonment, and drug and alcohol abuse (*5*). Drug users are commonly immunocompromised through HIV infection and malnutrition, resulting in increased risk for TB infection and rapid progression to active disease.

Habitually smoking crack cocaine causes pulmonary damage (crack lung) (Figure). Consequently, alveolar macrophage function and cytokine production is impaired, which may enhance susceptibility to infectious diseases (*6*). *Mycobacterium tuberculosis* is an intracellular pathogen that begins the disease process after a person inhales bacilli into the terminal bronchi and pulmonary alveoli (*7*). Alveolar epithelial cells likely resist invasion by *M. tuberculosis* bacilli, enabling resident alveolar macrophages and dendritic cells sufficient time to traverse the epithelium and phagocytose potential invading microbes (*8*). Several pulmonary complications are associated with the inhalation of crack cocaine (e.g., intensive cough, hemoptysis, shortness of breath, chest pain, acute bilateral pulmonary infiltrates, thermal airway injury, pneumothorax and noncardiogenic pulmonary edema, production of carbonaceous sputum, and exacerbation of asthma) (*9*). Collectively, these complications have been reported as crack syndrome (*10*). We hypothesize that crack cocaine use increases the risk for smear-positive pulmonary TB and that a component of this risk relates to lung damage caused by crack cocaine inhalation.

**Figure:**
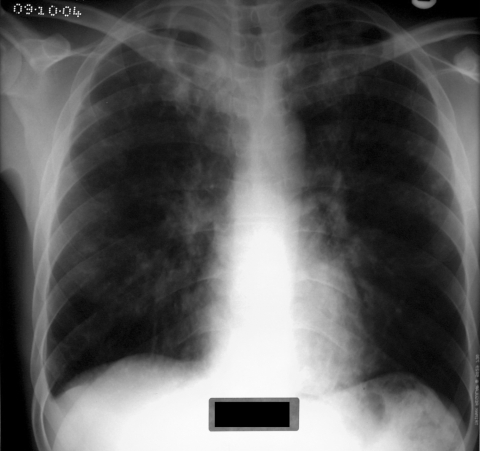
Chest radiograph of a tuberculosis patient addicted to crack cocaine.

## The Study

Detailed clinical and social data were collected by case managers for all TB patients undergoing treatment in London on July 1, 2003. The study was approved by the Metropolitan Multicentre Research Ethics Committee–United Kingdom. Analyses were restricted to pulmonary patients 15–60 years of age (n = 970). We used univariate analyses to compare the characteristics of crack cocaine users, other hard-drug users (predominantly heroin users but excluding those who used only alcohol and marijuana), and those not known to use drugs. A separate category was included for hard-drug users not known to use crack cocaine to have a group with comparable levels of social deprivation, addiction related problems, and difficulty in accessing health services. To test the hypothesis that smear positivity at diagnosis was associated with crack cocaine use, we used a multivariate model with backwards elimination to exclude variables that did not make a significant contribution to the model. Variables initially included are shown in Table 1; the final model is shown in Table 2.

**Table 1:** Univariate analysis of drug-using and non–drug-using patients with pulmonary TB in London, United Kingdom, 2003–2004*

Variable	No known drug use, n = 833, no. (%)	Hard-drug user (unconfirmed crack cocaine user), n = 115, no. (%)	Hard-drug user (confirmed crack cocaine user), n = 22, no. (%)	p value
Gender				<0.0001
Male	445 (54.1)	99 (86.8)	12 (54.6)	
Female	377 (45.9)	15 (13.2)	10 (45.5)	
Ethnicity				<0.0001
White	142 (17.1)	54 (47.0)	5 (22.7)	
Black African	344 (41.5)	25 (21.7)	5 (22.7)	
Black Caribbean	32 (3.9)	15 (13.0)	9 (40.9)	
South Asian	244 (29.4)	17 (14.8)	0	
Other	68 (8.2)	4 (3.5)	3 (13.6)	
Born in the United Kingdom	162 (19.6)	62 (54.9)	14 (63.7)	<0.0001
Previous TB	78 (9.4)	25 (21.7)	5 (22.7)	<0.0001
Previous TB past 2 years (relapsed)	36 (4.3)	19 (16.5)	4 (18.2)	<0.0001
Known HIV+	95 (11.4)	9 (7.8)	3 (13.6)	0.478
Delay in diagnosis >3 mo	109 (13.1)	19 (16.5)	2 (9.1)	0.499
Sought treatment at ED	126 (15.1)	32 (27.8)	10 (45.6)	<0.0001
Cough during initial examination	589 (70.7)	99 (86.1)	19 (86.40	0.001
Sputum smear positive at diagnosis	302 (36.3)	68 (59.1)	19 (86.4)	<0.0001
MDR	32 (3.8)	7 (6.1)	0	0.333
Linked to known INH resistance outbreak	9 (1.1)	10 (8.7)	11 (50.0)	<0.0001
INH resistance (not outbreak)	54 (6.5)	11 (9.6)	1 (4.5)	0.783
Treated with DOT from start	74 (9.0)	19 (16.5)	6 (27.3)	0.001
Nonadherent to treatment in first 2 mo	125 (15.0)	59 (51.3)	15 (68.2)	<0.0001
Lost to follow-up	19 (2.3)	12 (10.4)	6 (27.3)	<0.0001
Homeless	37 (4.4)	22 (19.1)	13 (59.1)	<0.0001
Mental health problems	28 (3.4)	27 (23.5)	9 (40.9)	<0.0001
Imprisoned during current episode of TB	9 (1.1)	22 (19.1)	14 (63.6)	<0.0001

*TB, tuberculosis; ED, emergency department; MDR, multidrug resistant; INH, isoniazid; DOT, directly observed therapy.

**Table 2:** Multivariate analysis of risk factors for smear-positive disease on diagnosis among drug-using and non–drug-using patients with pulmonary TB in London, UK, 2003–2004*

Variable	OR	95% CI	p value
Not a hard-drug user	Baseline		
Hard-drug user (not known to use crack cocaine)	1.87	1.19–2.95	0.007
Crack cocaine user	6.59	1.78–24.31	0.005
Age, y			
0–14	0.10	0.08–0.56	0.002
15–29	1.10	0.81–1.48	0.55
30–59	Baseline		
>60	0.69	0.45–1.14	0.14
Ethnicity			
South Asian	Baseline		
Black African	1.75	0.96–1.95	0.08
White	1.51	0.99–2.31	0.053
Black Caribbean	2.70	1.34–5.43	0.005
Other ethnicity	1.61	0.91–2.85	0.101
No drug resistance	Baseline		
INH (not outbreak strain)	1.23	0.72–2.11	0.441
INH (outbreak strain)	0.96	0.37–2.50	0.929
MDR	2.90	1.44–5.78	0.003
Sought treatment at ED	3.33	2.20–4.82	<0.001

*OR, odds ratio; CI, confidence interval; INH, isoniazid resistant; MDR, multidrug-resistant; ED, emergency department.

TB patients who used crack cocaine were predominantly 20–49 years of age. Crack cocaine users and other drug users were significantly more likely than non–drug users to have been born in the United Kingdom, of white or black Caribbean ethnic origin, homeless, alcohol abusers, or have a history of imprisonment. Non–crack drug users tended to have the longest delays between diagnosis and treatment and crack users the shortest, but this tendency did not reach significance (Table 1). Crack cocaine users were statistically significantly more likely to seek treatment at emergency departments, to adhere poorly to treatment regimen, or default from treatment altogether. Drug users were also more likely to have isoniazid-resistant disease. Among crack cocaine users this was primarily related to a large outbreak of isoniazid-resistant TB (*11*).

Among crack cocaine users, diagnosis showed that 86% were smear positive compared with 36% of patients not known to use drugs (relative risk [RR] 2.4, 95% confidence interval [CI] 2.0–2.9), p<0.001) and 59% of drug users not known to use crack cocaine (RR 1.6, 95% CI 1.4–2.0, p<0.001). Multivariate analysis showed that the risk for smear-positive disease was higher for drug users than for those not known to use drugs (odds ratio [OR] 1.9, 95% CI 1.2–3.0, p = 0.007) and highest in crack cocaine users (OR 6.6, 95% CI 1.8–24.3, p = 0.005). Other significant risk factors for smear positivity were being of black Caribbean ethnicity, having multidrug-resistant disease, and seeking treatment at an emergency department. When the multivariate model was restricted to include only hard-drug users, crack cocaine users were still significantly more likely than other drug users to be smear positive (p = 0.02).

## Conclusions

Smear-positive disease is 2.4 times more likely to be diagnosed in crack cocaine users than in non–drug users, whereas hard-drug users not known to use crack cocaine are 1.6 times more likely to be diagnosed with smear-positive disease. Crack cocaine users were significantly more likely than other drug users to be smear positive on diagnosis.

The increased risk for smear-positive disease in crack cocaine users was not due to diagnostic delays. Hard-drug users who were not confirmed as crack cocaine users had the longest diagnostic delays. Crack cocaine users had the shortest diagnostic delays, potentially attributable to rapidly progressive, debilitating disease. Crack cocaine users were also more likely to seek treatment at an emergency department rather than primary care services. Again, the choice of healthcare service may be related to the severity of disease. Symptom duration before diagnosis is difficult to measure, especially among drug users. We included non–crack drug users as a comparison group because they have a similar social profile and similar access to healthcare. Therefore, we are confident that the extremely high levels of smear positivity on diagnosis in crack cocaine users are not due to a long duration of clinical illness preceding diagnosis.

In the multivariate model, crack cocaine use remains strongly associated with smear-positive disease after controlling for a wide variety of other potential confounders. Other risk factors include ethnicity (drug use was common among black Carribean patients and may have been underreported); treatment at an emergency department (possibly a marker of disease severity); and multidrug-resistant disease. We are uncertain why multidrug-resistant cases were more likely to be smear-positive on diagnosis; however, previous studies have found that cavitary disease is a risk factor for drug resistance (*12*).

 The fact that smear positivity was significantly more prevalent in patients known to use crack cocaine when compared with other hard-drug users suggests that this additional risk may be attributable to a biological component. Plausible biological mechanisms to explain the increased risk of smear-positive disease include poor alveolar macrophage antimicrobial activity in crack cocaine users due to decreased inducible nitric oxide synthase activity (*13*) and direct effects on the lung (*10*).

It is likely that a proportion of hard-drug users were incorrectly classified as not using crack cocaine due to nondisclosure. This would reduce the apparent differences in levels of smear positivity between the groups. Nevertheless, despite relatively small numbers of known crack cocaine users, there is a significantly (p = 0.02) higher proportion of smear-positive disease in these patients compared with other hard-drug users.

Previous studies have shown TB transmission associated with crack cocaine use (*14*). Persons frequenting crack houses are likely to have multiple risk factors for active pulmonary TB. Prolonged sharing of closed and confined airspace, intensive coughing, and other acute pulmonary complications of crack cocaine inhalation promote transmission. Drug users are more likely than non–drug users to default treatment, to remain infectious for prolonged periods after diagnosis, and to acquire drug-resistant TB (*15*). We studied smear status at diagnosis to exclude the effect of poor treatment adherence.

Our study suggests a dangerous synergy between TB and crack cocaine. Users may experience addiction-related problems that complicate access to healthcare and aggravate transmission, possibly aggravated by a biological driver that may increase susceptibility to infection and progression to infectious disease. Additional studies are needed to investigate the possible biological role of crack cocaine in the development of infectious forms of TB.
